# Development of a Current Collector with a Graphene Thin Film for a Proton Exchange Membrane Fuel Cell Module

**DOI:** 10.3390/molecules25040955

**Published:** 2020-02-20

**Authors:** Yean-Der Kuan, Ting-Ru Ke, Jyun-Long Lyu, Min-Feng Sung, Jing-Shan Do

**Affiliations:** 1 Department of Refrigeration, Air-Conditioning and Energy Engineering, National Chin-Yi University of Technology, Taichung City 41170, Taiwan; muke1002@gmail.com (T.-R.K.); hyt5406@gmail.com (J.-L.L.); 2 Kenda Rubber Ind. Co., Ltd., Yuan-Lin 51064, Taiwan; song221@gmail.com; 3 Department of Chemical and Materials Engineering, National Chin-Yi University of Technology, Taichung City 41170, Taiwan; jsdo@ncut.edu.tw

**Keywords:** proton exchange membrane fuel cell, graphene thin film, current collector, module

## Abstract

This paper constructs planar-type graphene thin film current collectors for proton exchange membrane fuel cells (PEMFCs). The present planar-type current collector adopts FR-4 as the substrate and coats a copper thin film using thermal evaporation for the electric-conduction layer. A graphene thin film is then coated onto the current collector to prevent corrosion due to electrochemical reactions. Three different coating techniques are conducted and compared: Spin coating, RF magnetron sputtering, and screen printing. The corrosion rates and surface resistances are tested and compared for the different coating techniques. Single cell PEMFCs with the developed current collectors are assembled and tested. A PEMFC module with two cells is also designed and constructed. The cell performances are measured to investigate the device feasibility.

## 1. Introduction

The proton exchange membrane fuel cell (PEFMC) adopts hydrogen as its fuel, and the fuel energy is converted into electrical energy through an electrochemical reaction. In portable applications, bipolar plates or current collectors account for the major weight percentage among PEMFC components [1]. In portable applications, the PEMFC power requirement is merely a few watts. The PEMFC Balance of Plant (BOP) consumption should be as low as possible; therefore, the PEMFC planar-type module could be more suitable than the conventional, vertical PEMFC stack, as it could omit the air fan or air-pump at the cathode side via the self-air breathing design, significantly reducing the device thickness.

In order to shrink the fuel cell size for portable applications, Lee et al. first proposed the micro-electro-mechanical system (MEMS) technique to construct a micro fuel cell [2]. After that, MEMS techniques were intensively investigated for micro fuel cell fabrication. MEMS techniques were applied in planar-type current collector fabrications, such as the metal lift-off process [3], metal powers deposition onto the wafer surface [4], coating gold-titanium and gold-nickel onto a stainless steel thin plate [5], coating Au onto 316L stainless steel mesh via electro-deposition [6], coating TiN, TiAlN mono-layers, and TiN/TiAlN bi-layers onto 316L stainless steel plates via the physical vapor deposition (PVD) process [7]. The application of MEMS techniques to fabricate micro channels for micro fuel cells was also widely studied, including micro channel fabrication onto a silicon substrate [8], constructing micro channels and metallization onto a polymethyl methacrylate (PMMA) substrate [9], or adopting the electroforming process to make micro channels on a thin copper substrate [10].

Although good electrical conductivity is important for micro fuel cell current collectors, corrosion resistance is also very important, as poor corrosion resistance degrades micro fuel cell durability. Related research has been conducted on current collector electrical conductivity, such as coating gold or TiN/TiAlN layers onto stainless steel expanded meshes [11], depositing a NieP (10e12%P) thin layer onto aluminum thin sheets [12], and coating Ag Nano wires with polydimethylsiloxane (PDMS), as current collector flow channels [13,14].

The printed-circuit board (PCB) technique was also applied to construct the planar-type portable fuel cell, which was first proposed by O’Hare et al. [15] and Schmitz et al. [16]. Some related studies were conducted using flexible PCB as a current collector for air-breathing planar fuel cell stacks [17], process-induced fuel cell damage due to the PCB assembly process [18], and a disc type current collector with copper clad aluminum surface coated with gold using the PCB process [19].

To avoid distortion due to the mismatch of significantly different material properties between the metal current collector and FR-4 substrate in the PCB fuel cell, a lightweight current collector was proposed by Sung et al. [20] and Kuan et al. [21], coated with a copper thin film for electric conduction and a nickel thin film for corrosion resistance, on a FR-4 plate as the substrate, via the thermal evaporation technique. The authors’ research team subsequently coated a graphene thin film instead of a nickel thin film as the corrosion-resistance layer. The graphene suspension was adopted and coated onto to the current collector electric-conduction layer via the spinning coating and vacuum oven processes.

A single cell PEMFC was also assembled and tested [22]. Ning et al. [23] proposed a light and flexible air breathing PEMFC using a carbon nanotube (CNT) membrane with holes as the current collectors, to form flexible composite electrodes. They also discovered that thicker carbon nanotube membranes would show higher cell performance especially for large size flexible power sources. In addition, adjusting the directions of the current collectors could also significantly increase the cell performance due to the proper electron transfer pathways that might effectively reduce electric resistance [24]. Mallick et al. [25] made a critical review of current collectors for passive direct methanol fuel cell (DMFC). They indicated that the open ratio of current collector is a major parameter of the DMFCs, the reduction of weight is valuable to increase the gravimetric energy density, and the metal mesh and porous metal are potential substitutes to the perforated plate current collectors in the passive DMFC.

In addition, the precious metal coating of the current collector is essential for improving corrosion resistance; however, it would increase the total cost of the cell. Optimizing the coating thickness is essential to maintain the balance between the corrosion resistance and cost. Surfaces and interfaces present a significant portion of the workable area and a network of energetically mismatched or metastable molecules, which could be exploited to either control surface reactions, engineer bulk stability, or reveal new fundamental details of those now well understood processes or systems [26]. Lee et al. [27] adopted chemical vapor deposition (CVD) to produce graphene coated bipolar plates. The experimental results show that the ultra-thin graphene layer on the copper bipolar plate could act as a very thin passivation layer that could minimize the surface oxidation on the copper plate without performance degradation of the polymer electrolyte fuel cells.

The main objective of this paper is to develop lightweight planar-type current collectors for a PEMFC module with two cells. The lightweight current collector also adopts FR-4 as the substrate material; the copper thin film is coated via the thermal evaporation process that functions as the electric-conduction layer. A graphene thin film is further coated onto the copper thin film as the corrosion-resistance layer. The proposed planar-type current collectors were fabricated with three different graphene thin film coating processes. The completed current collectors were assembled into a two-cell PEMFC module. Related performance and stability experiments were also performed.

## 2. The Lightweight Planar-Type Current Collector Construction

The present lightweight current collector for the two-cell PEMFC module consists of three layers. The first layer is the FR-4 substrate. The second layer is the electric-conduction layer–copper thin film fabricated by adopting the thermal evaporation process to coat copper particles onto the FR-4 surface. The third layer is the corrosion-resistance layer–graphene thin film coating via three different processes discussed and compared in this paper.

**Figure 1:**
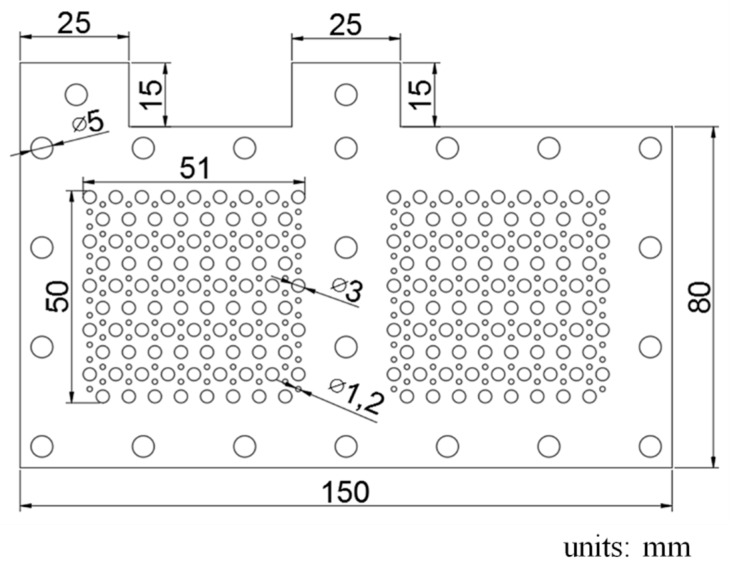
Geometric schematic drawing of the current collectors.

A geometric drawing of the two-cell current collector is shown in Figure 1. The current collector outline is 150 × 80 mm and the reaction area of each membrane electrolyte assembly (MEA) is 50 × 50 mm. Holes of two diameters, 3 and 1.2 mm, are arrayed in the reaction area. Two 25 × 15 mm cuboids for in/out are connected among the electric cells.

**Figure 2:**
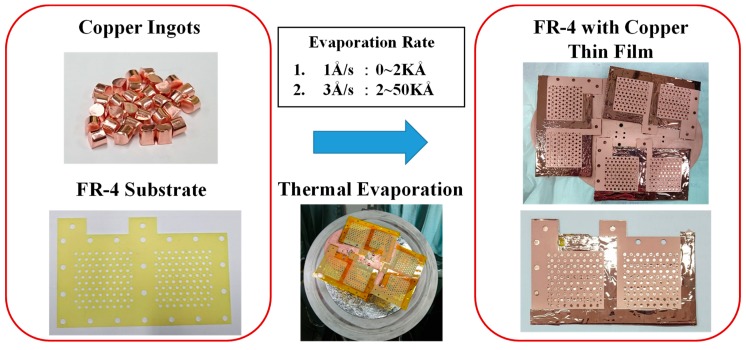
Fabrication process for the electric-conduction layer.

The fabrication process for the electrical conduction layer is shown in Figure 2. The machined FR-4 substrate has a heat-resistant tape pasted onto the back side with copper ingots placed into the evaporator vacuum chamber. Once the chamber is vacuumed to 5E-5 torr pressure, the thermal evaporation process begins, and the copper ingots are evaporated and then coated onto the FR-4 substrate to a 50 Åm thickness. The evaporation rate is controlled at 1 Åm/s in the 0–2 kÅm evaporation thickness range and increased to 3 kÅm in the 2–50 kÅm evaporation thickness range. When the copper thin film is completely coated as the electrical conduction layer, the graphene thin film coating as the corrosion-resistance layer is then added. Three different types of graphene thin films are investigated, graphene ink, graphene suspension, and graphene dispersion.

**Figure 3:**
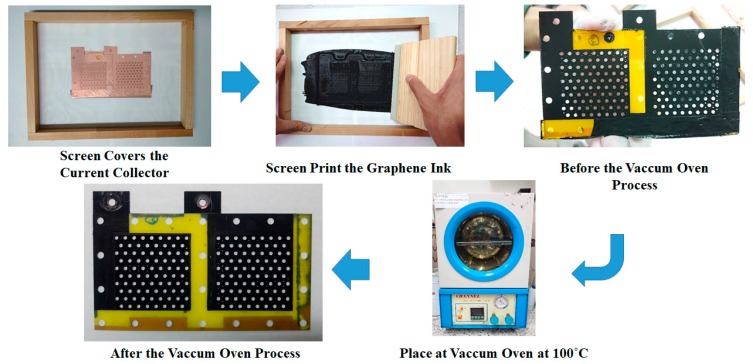
Fabrication process for a graphene thin film using graphene ink.

The fabrication process for the graphene thin film using graphene ink is shown in Figure 3. A screen is placed to cover the FR-4 current collector substrate with copper thin film. The graphene ink is then coated onto the surface through screen printing. Then, the current collector with the graphene thin film is placed into the vacuum oven, the vacuum degree is 76 cm Hg, and the oven temperature is kept at 100 °C for 30 min to evaporate the water contained in the graphene thin film. Additionally, the vacuum temperature is reduced to room temperature and the completed current collector is removed from the oven. The graphene ink adopted in this paper is a commercial graphene ink produced by Enerage Inc. The appearance is a black paste, the adhesion is larger than 4B (for PET film), the pencil hardness is larger than HB, the sheet resistance is less than 1968.5 Ω/sq/mm, and the viscosity is 35,000 ± 10,000 cP [28].

**Figure 4:**
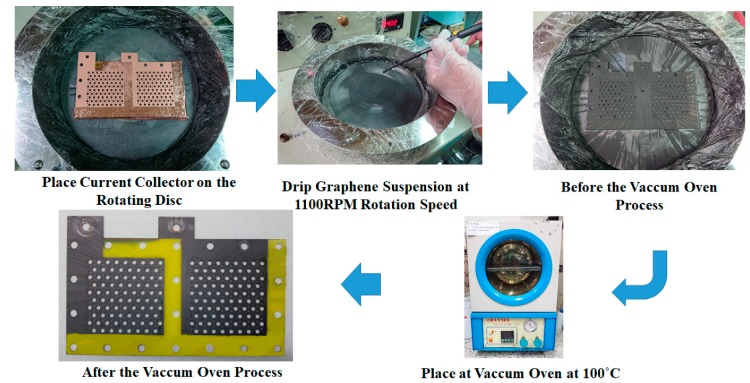
Fabrication process for a graphene thin film using graphene suspension.

The graphene thin film fabrication process uses a graphene suspension, as shown in Figure 4. The current collector FR-4 substrate has a precoated copper thin film on the surface produced by a spin coater rotating disc at 1100 rpm for 60 s. A 9 mL graphene suspension is slowly dripped onto the FR-4 copper thin film substrate. Then, the current collector with the graphene thin film is placed into a vacuum oven. The vacuum degree is 76 cm Hg and the oven temperature is kept at 100 °C for 30 min to evaporate the water contained in the graphene thin film. The vacuum temperature is then reduced to room temperature with the complete current collector removed from the oven. The graphene suspension adopted in this paper is a commercial graphene suspension by Enerage Inc., the appearance is a gray black liquid, and the solvent is water. The solid content is 5 wt% and the additive is less than 2 wt%. The graphene suspension viscosity is 2500 ± 500 cP. The PH value is approximately 8.0. The average lateral size is less than 15 μm, and the fineness is 5 μm [29].

**Figure 5:**
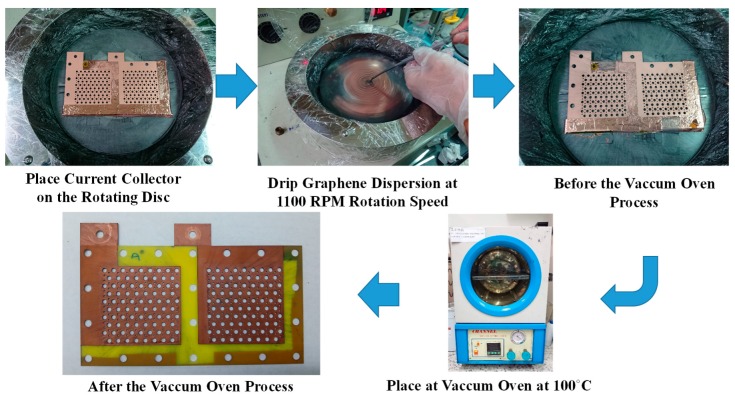
Fabrication process for a graphene thin film using graphene dispersion.

The graphene thin film fabrication process using graphene dispersion is shown in Figure 5. The current collector FR-4 substrate with a precoated copper thin film on the surface is placed on a spin coater rotating disc at 1000 rpm for 60 s. The 6 mL graphene dispersion is slowly dripped onto the FR-4 substrate copper thin film. Then, the current collector with the graphene thin film is placed into a vacuum oven at 100 °C for 30 min to evaporate the dispersant contained in the graphene thin film. The vacuum temperature is reduced to room temperature and the complete current collector is removed from the oven. The graphene dispersion adopted in this paper is a commercial graphene suspension produced by GI Business. The liquid is black and adapts EAC (ethyl acetate) as the dispersant. The graphene sheets are 0.1–20 μm in diameter, with a less than 3 nm average thickness, and a concentration larger than 5000 ppm [30]. As the graphene dispersion is very dilute, the graphene film spin coating is ultra-thin; therefore, although the surface color does not change distinctly, the ultra-thin film could be a very thin passivation layer on the copper thin film, which would minimize the surface oxidation [27].

## 3. Results and Discussion

The measurements on the weight and thickness of the current collectors are shown in Table 1. The thickness and weight of the FR-4 substrate of the current collector are 0.5 mm and 9.015 g, respectively. After coating the copper thin film, the thickness and weight are 0.51 mm and 9.134 g, respectively. The increment of the thickness after further coating of the graphene is very small. For both the graphene suspension and graphene coated with thin film, the total thickness is not changed via the Vernier caliper measurements. Only the graphene ink coating could be measured at the 0.01 mm thickness increment. After coating, the current collector with the graphene ink thin film shows the highest and most significant weight increment, followed by the current collector with the graphene suspension thin film; the current collector with the graphene-dispersion thin film has the lowest weight increment. Therefore, it can be inferred that the graphene ink thin film has the largest thickness, the graphene suspension has a medium thickness, and the graphene-dispersion thin film has the smallest thickness.

**Figure 6:**
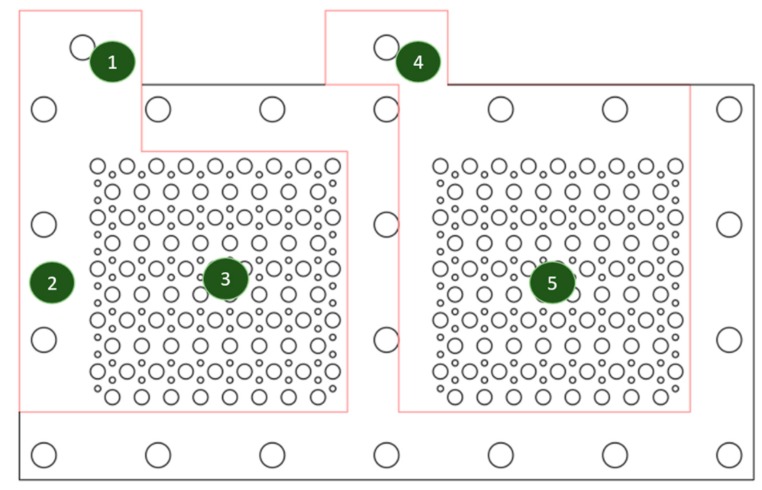
The illustration of five monitoring points for the surface resistance.

The four probe resistance measurements were conducted to ensure electrical conductibility uniformity for the complete current collectors. An illustration of the five current collector monitoring points for surface resistance measurements is shown in Figure 6.

The current collector surface resistance values with a graphene thin film using graphene ink, graphene suspension, or graphene dispersion, for the complete current collectors, are shown in Table 2, Table 3 and Table 4, respectively. The results show that all surface resistances are low, and the values for the current collector with a graphene thin film using graphene ink are in the range between 11.19 and 15.40 mΩ/sq. The values for the current collector with a graphene thin film using graphene suspension are in the range between 11.55 and 19.12 mΩ/sq. The values for the current collector with a graphene thin film using graphene dispersion are in the range between 11.33 and 17.58 mΩ/sq, which also shows that the coating is quite uniform in all three processes. In addition, the surface resistance values before and after coating the current collector with a copper thin film and graphene thin film are quite close in all three processes. This implies that the corrosion-resistance layer would not reduce the electrical conduction capability.

**Figure 7:**
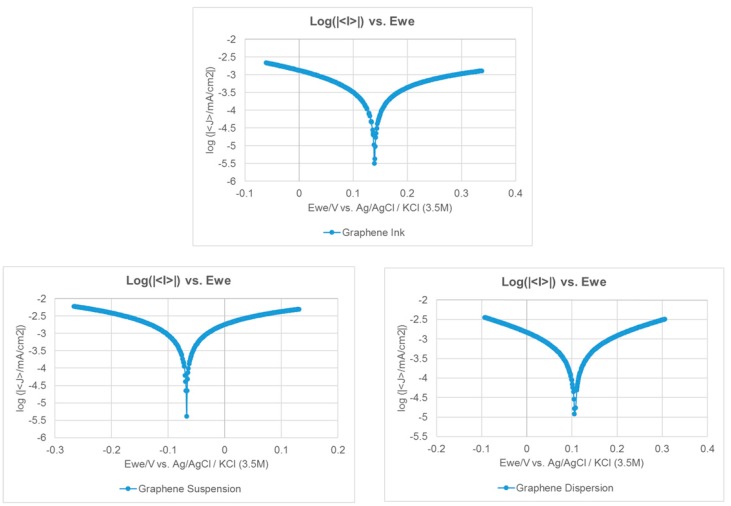
The Tafel curves of the current collectors with different graphene films.

Preventing corrosion is also very important for the current collectors. Therefore, the Tafel extrapolation method is conducted to measure the corrosion characteristics. An aqueous methanol solution and de-ionized water are first prepared and mixed as an acidic aqueous methanol solution, the mix ratio of methanol to de-ionized water is 1:9 and the PH value is 6.2. A sample of the fabricated current collector would then be placed in the solution. The Ag/AgCl (3.5M KCl) is adopted as the reference electrode with a platinum counter electrode. Impedance measurements were conducted adopting the BioLogic SP-150AC instrument. The scan rate is 0.166 mV/s and the voltage is −0.025–0.025 V. The results of the Tafel curves are shown in Figure 7, the corrosion voltage (Ecorr) and corrosion current (Icorr) results are shown in Table 5. The higher corrosion resistance would show a higher Ecorr value and the higher corrosion rate would show a higher Icorr value. The results show that adopting the graphene dispersion to fabricate the current collector presents the highest corrosion resistance, adopting the graphene ink second, and adopting the graphene suspension presents the lowest corrosion resistance.

**Figure 8:**
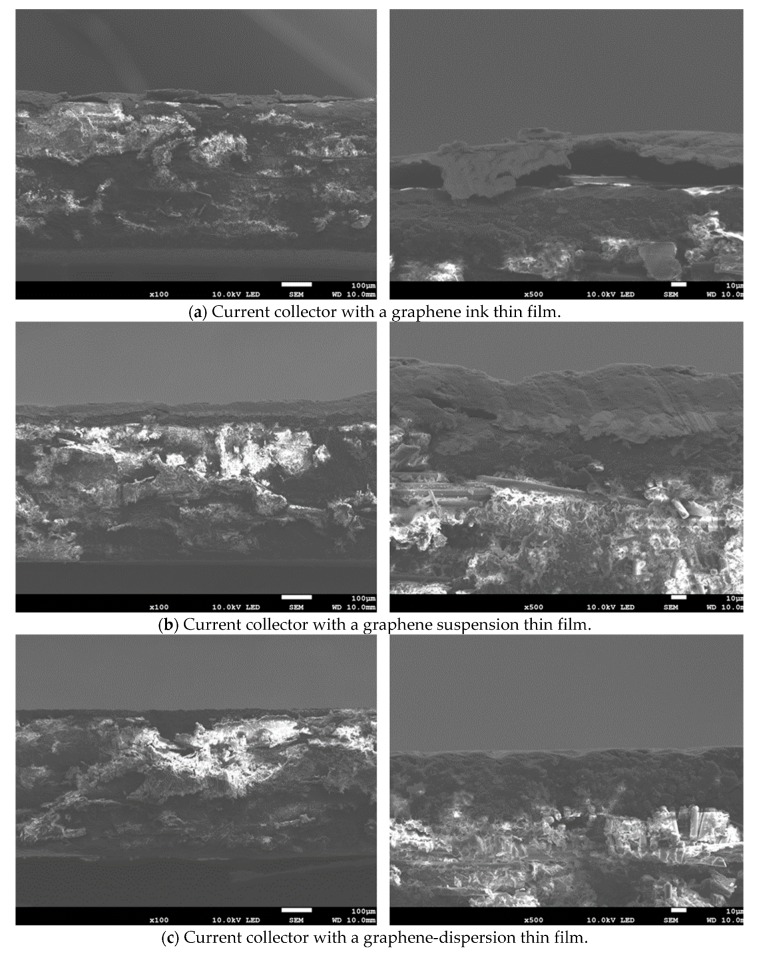
The scanning electron microscopy (SEM) images of the cross sections of the current collectors with different graphene thin films. (**a**) Graphene ink; (**b**) Graphene suspension; (**c**) Graphene dispersion.

Scanning electron microscopy (SEM) is adopted to investigate the micrographs of the cross-sections of the current collectors coated with different graphene thin films, and this is shown in Figure 8a–c. The graphene ink thin film is the most uneven at the surface and there are some obvious voids inside, the graphene suspension thin film is smoother at the surface with much fewer voids inside, and the graphene-dispersion thin film is the thinnest and smoothest at the surface.

After completed current collector fabrication, the proposed current collectors were assembled into a two-cell PEMFC module to measure cell performance. PEMFC module with both forced convection air-breathing cathodes and self-air-breathing cathodes were studied. The MEA adopted in this paper is a three-layer catalyst coated type with a DuPont Nafion HP membrane and carbon paper diffusion layers. The reaction area was 5 × 5 cm, the catalyst load was Pt 0.1 mg cm^−2^ at the anode, and Pt 0.4 mg cm^−2^ at the cathode. Silicon gaskets with a 0.15 mm thickness were used for sealing. The cell compression ratio was 34.78%.

**Figure 9:**
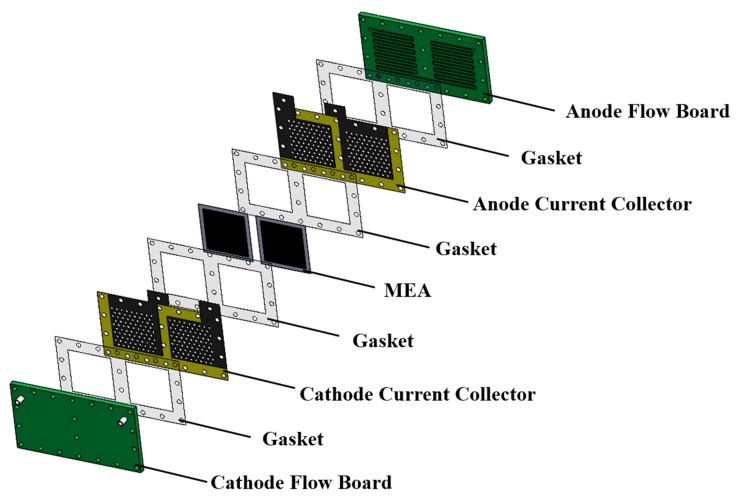
The exploded view of the Proton Exchange Membrane Fuel Cell (PEMFC) with forced convection air-breathing cathode. Membrane electrolyte assembly (MEA).

**Figure 10:**
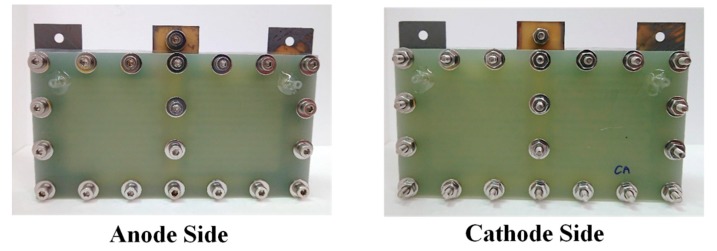
The anode side and cathode side of the PEMFC module with a forced convection air-breathing cathode.

**Figure 11:**
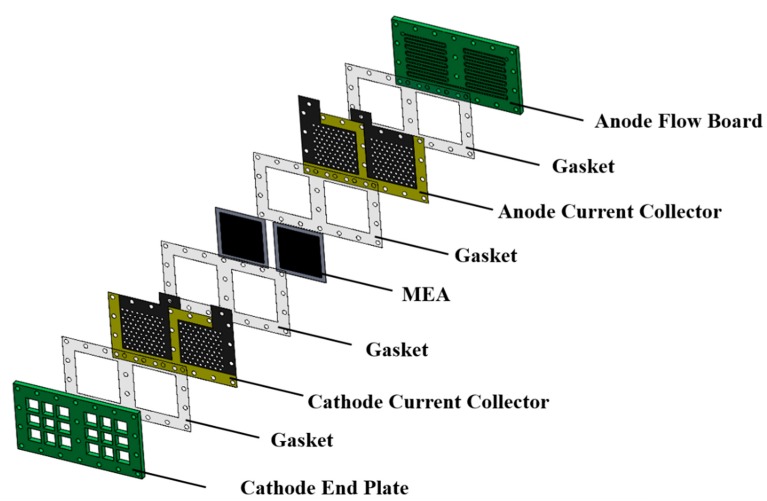
The exploded view of the PEMFC module with a self-air-breathing cathode.

**Figure 12:**
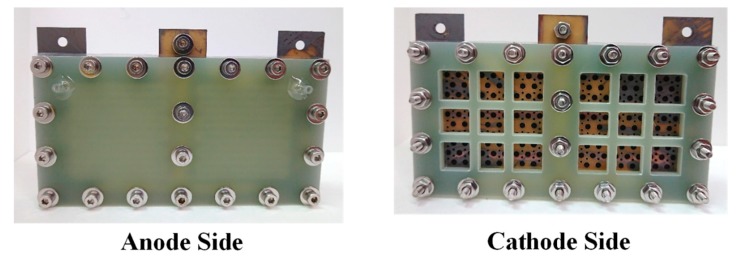
The anode side and cathode side of PEMFC module with a self-air breathing cathode.

An exploded drawing of the two-cell PEMFC module with a forced convection air-breathing cathode is shown in Figure 9. From cathode to anode, the components are cathode flow board, gasket, cathode current collector, gasket, MEA, gasket, anode current collector, gasket, and anode flow board, respectively. A picture of the assembled PEMFC module with a forced convection air-breathing cathode is shown in Figure 10. An exploded drawing of the two-cell PEMFC module with a self-air-breathing cathode is shown in Figure 11. From cathode to anode, the components are cathode end plate, gasket, cathode current collector, gasket, MEA, gasket, anode current collector, gasket, and anode flow board, respectively. A picture of the assembled PEMFC module with self-air-breathing cathode is shown in Figure 12.

The PEMFC module performances with forced convection air-breathing and three different types of graphene coatings were conducted at different anode hydrogen and cathode air flowrates. The anode hydrogen flowrate and cathode air flow rate ratios were kept at 1:2 in the experiment. Both the anode hydrogen flow and cathode airflow were humidified and kept at 50 °C. The anode hydrogen/cathode air flow rates of 25/50, 50/100, 100/200, 200/400, 300/600, and 400/800 sccm were investigated.

**Figure 13:**
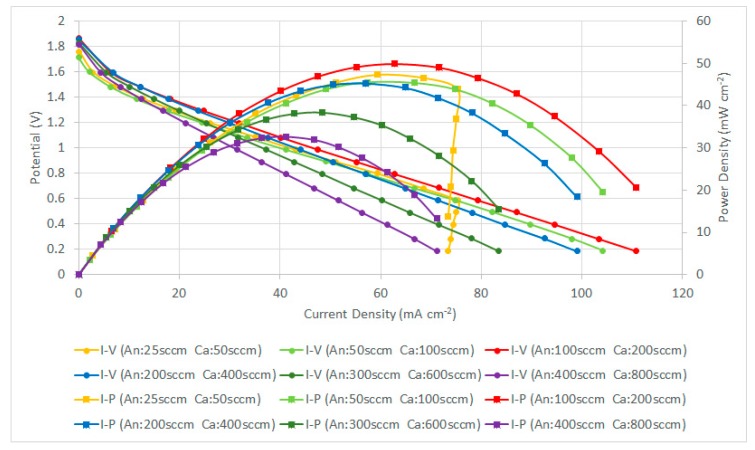
PEMFC module performance comparison with a forced air-breathing cathode and the corrosion-resistance layer using a graphene ink coating.

A performance comparison of the PEMFC module with a forced air-breathing cathode and the corrosion-resistance layer using graphene ink coating is shown in Figure 13. The results show that the PEMFC module performance increased with the increase in the low flow rate and reached the highest performance at the anode/cathode flow rates of 100/200 sccm. At low flow rates, increasing the flow rate is helpful to decrease the low mass transfer effect that would increase the cell performance. Whereas at high flow rates, increasing the flow rate reduced the cell performance, due to sub-saturated streams, which dehydrates the membrane, which is caused by the cell temperature inside exceeding the dew point. 

In the high-current range of higher than 75 mA cm^−2^ at the low flow rate (25/50 sccm), the further reduction of the operating voltage leads to a sharp drop of the current. This is due to the insufficient reactant, and the fuel cell becomes highly unstable, the cell performance is sharply dropped and is without a smooth transition through the ohmic loss to the concentration loss. Therefore, the low flow rate condition is not suitable for the cell operation at the high-current range for the current collector with the graphene ink thin film.

**Figure 14:**
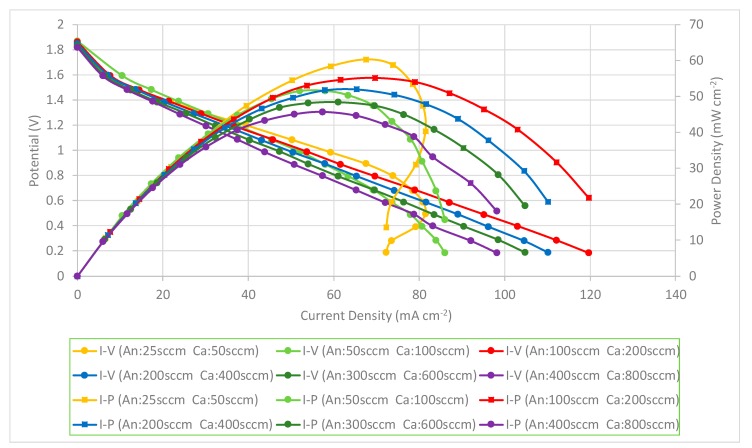
PEMFC module performance comparison with a forced air-breathing cathode and the corrosion-resistance layer using a graphene-suspension coating.

The PEMFC module performance is decreased when the flow rates are increased. The PEMFC module performance comparison with a forced air-breathing cathode and a corrosion-resistance layer using a graphene-suspension coating is shown in Figure 14. The results also show that the PEMFC module performance is increased at the low flowrate and reaches the highest performance at the anode/cathode flow rates of 100/200 sccm. The PEMFC module performance is decreased when the flow rates are further increased. At low flow rates, increasing the flow rates is helpful to decrease the low mass transfer effect that increases the cell performance. The results show a similar trend as the current collectors with graphene ink coatings and the related explanation refers to the Figure 13 explanation. 

**Figure 15:**
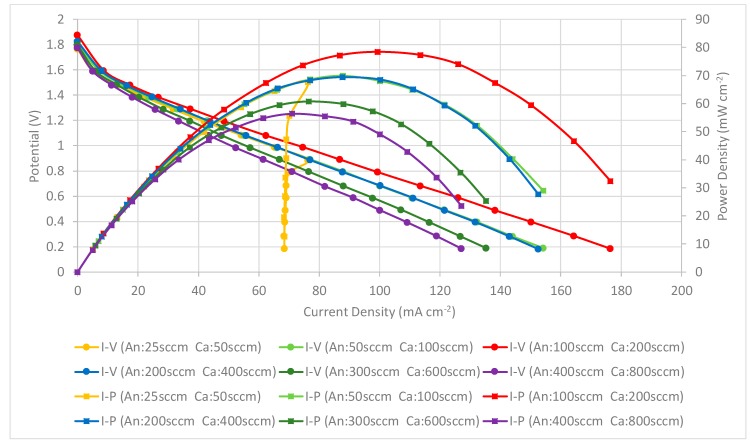
PEMFC module performance comparison with a forced air-breathing cathode and the corrosion-resistance layer using a graphene-dispersion coating.

The performance comparison of the PEMFC module with a forced air-breathing cathode and the corrosion-resistance layer using a graphene-dispersion coating is shown in Figure 15. Similar to the previous two cases, the results still show that the PEMFC module performance is increased when increasing at the low flow rate and reaches the highest performance at the anode/cathode flow rates of 100/200 sccm. The performance comparison of the PEMFC module with a forced air-breathing cathode and a corrosion-resistance layer, at the anode/cathode flow rates is 100/200 sccm. At low flow rates, increasing the flow rates is helpful to decrease the low mass transfer effect that can increase the cell performance. Whereas at high flow rates, increasing the flow rate would reduce the cell performance due to sub-saturated streams, which dehydrates the membrane, and is caused by the cell temperature inside exceeding the dew point. In the high-current range of higher than 75 mA cm^−2^ at the low flow rate (25/50 sccm), the further reduction of the operating voltage will lead to a sharp drop of the current. The results also show a similar trend to the current collectors with a graphene ink coating and the related explanation refers to the Figure 13 explanation. 

The results show that the current collectors with three different types of graphene thin film show similar phenomena and trends. In addition, the current collector using the graphene-dispersion coating presents the highest performance, using graphene suspension is second, and using graphene ink presents the lowest PEMFC module performance.

**Figure 16:**
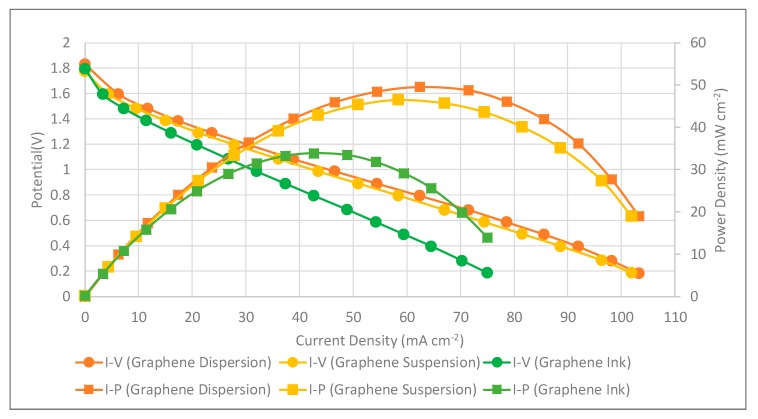
PEMFC module performance comparison with a self-air-breathing cathode and different types of the corrosion-resistance layers (anode fuel rate: 100 sccm).

The PEMFC module performances with self-breathing and three different types of graphene coatings were further conducted at the anode hydrogen flow rate of 100 sccm. The anode hydrogen flow was humidified, kept at 50 °C and a 100 sccm flow rate. The cathode side was open for self-air-breathing and the ambient temperature was 25 °C. The performance comparison of the PEMFC module with self-air-breathing cathode and the corrosion-resistance layer is shown in Figure 16. Similar to the force convection air-breathing cathode, the results also show that the current collector using graphene-dispersion coating presents the highest performance, using graphene suspension second, and using graphene ink presents the lowest PEMFC module performance. Cell performance of the self-air breathing cathode is lower than the forced air-breathing. This is due to the lower air stoichiometry, a lower temperature caused by the lower room temperature would reduce the kinetic activation, reduce the mass transfer, and increase the ohmic loss.

Although the surface resistances are roughly the same for all cells via different graphene thin film coatings in Table 2, Table 3 and Table 4, there are still differences in the polarization resistances at the ohmic regime. This implies the surface resistance, which reflects the in-plane resistance, might not play the most important role. In the design concept, the graphene thin film is a corrosion-resistance layer, which is coated on the copper thin film layer, which functions as the electric-conduction layer. Therefore, the thinner the graphene layer, the better, and the interface between the graphene thin film and copper layer will also likely be better. If the interface is not good, it might degrade the electric conductivity. 

According to the measurements in Table 1 and the SEM images in Figure 8, the thickness and weight of the graphene ink thin film is significantly larger than the other two thin films, with some obvious voids inside the graphene ink thin film, and the surface of the graphene ink thin film is much more uneven than the other two thin films. The graphene-dispersion thin film has the lowest weight and the most flatness. The graphene-dispersion thin film has a little bit higher weight and the surface is uneven. The above could be why the current collector with the graphene ink thin film shows the significantly lowest performance, the performances of the other two are close to each other; however, the current collector with the graphene-dispersion thin film has the best performance overall.

**Figure 17:**
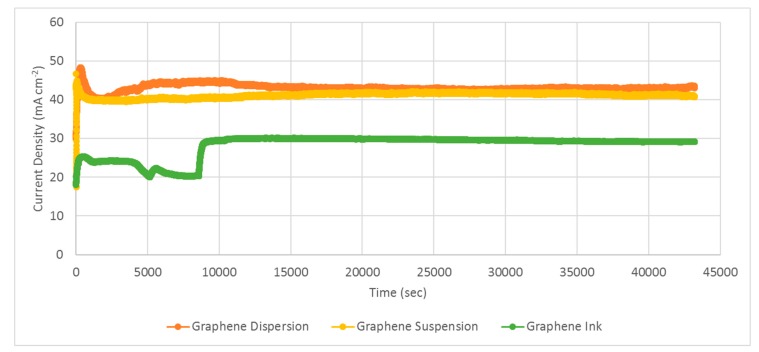
PEMFC module stability tests with a self-air-breathing cathode and different types of corrosion-resistance layers under 1 V load (anode fuel rate: 100 sccm).

After investigating the PEMFC module performance with the developed current collectors for both forced air-breathing and self-air-breathing cathodes, long-term stability tests of the PEMFC modules, with self-air-breathing cathodes, using three different graphene coatings as corrosion-resistance layers, were further conducted. Experiments were performed at a 100 sccm anode hydrogen flow rate and the hydrogen was humidified and kept at an inlet fuel temperature of 50 °C. The cathode side is open for self-air-breathing and the ambient temperature is 25 °C. The PEMFC module stability tests with self-air-breathing cathodes and different types of corrosion-resistance layers under 1 V loads, are shown in Figure 17. 

The results show that both the PEMFC modules with current collectors using graphene-dispersion and graphene-suspension coatings are quite stable. The PEMFC module performance with the collectors using the graphene-dispersion coating is slightly higher than that using the graphene suspension. Although the PEMFC module with current collectors using the graphene ink coating also showed good stability, the PEMFC module performance is distinctly lower than those using the graphene dispersion and graphene suspension. The results also successfully show the feasibility of the developed current collectors in portable applications. However, the tests only represent approximately a 10 h operation period, which is much less than the anticipated life specification. Therefore, additional longer duration tests, particularly under realistic cycling conditions that accelerate degradation, should be made in the future for realistic commercialized applications [31].

## 4. Conclusions

This paper proposes lightweight current collectors and constructed a PEMFC module with two cells. The current collectors consist of a FR-4 substrate, a copper thin film as the electric-conduction layer, and a graphene thin film as the corrosion-resistance layer. The copper thin film is accomplished by a thermal evaporation process. Three different graphene coating processes to fabricate the graphene thin film were investigated. The results show that adopting a graphene-dispersion coating has the best corrosion-resistance capability, the graphene-suspension coating is second, and graphene ink is the lowest. Further study of the fabricated current collectors assembled into a two-cell PEMFC module show that the current collectors with a graphene-dispersion coating have the highest PEMFC module performance, the graphene-suspension coating is second, and graphene ink also has the lowest PEMFC module performance. 

The graphene-dispersion thin film shows the lowest coating weight and superior surface flatness, and the graphene ink thin film shows a significantly higher coating weight, voids inside, and an uneven surface in the testing results. Long-time stability studies were also conducted, and all three graphene coating processes showed good PEMFC module stability after long-term operations. During the long-time stability tests, the PEMFC module performances for the current collectors that used a graphene-dispersion coating are a little bit higher than for the current collectors that used a graphene-suspension coating. These performances were both clearly higher than those of current collectors that used a graphene ink coating. The feasibility studies also showed the potential of the developed current collectors in portable applications.

## Figures and Tables

**Table 1:** Weight and thickness of the current collectors.

Item	FR-4 Substrate	Copper Thin Film Coated	Graphene Ink Thin Film Coated	Graphene Suspension Thin Film Coated	Graphene-Dispersion Thin Film Coated
Weight (g)	9.105	9.134	9.55	9.176	9.168
Thickness (mm)	0.5	0.51	0.52	0.51	0.51

**Table 2:** Surface resistances of a current collector with a graphene thin film using graphene ink.

	Monitoring Point	1	2	3	4	5	Standard Deviation (STD DEV)	Unit
Layers								
Copper Thin Film Layer		15.09	15.40	14.50	11.33	14.04	1.62	mΩ/sq
Graphene Thin Film Layer		15.27	15.31	15.40	11.19	14.23	1.79	mΩ/sq

**Table 3:** Surface resistances of a current collector with a graphene thin film using graphene suspension.

	Monitoring Point	1	2	3	4	5	STD DEV	Unit
Layers								
Copper Thin Film Layer		15.01	18.33	18.21	11.24	16.08	2.90	mΩ/sq
Graphene Thin Film Layer		15.49	19.12	18.67	11.55	16.04	3.03	mΩ/sq

**Table 4:** Surface resistances of a current collector with a graphene thin film using graphene dispersion.

	Monitoring Point	1	2	3	4	5	STD DEV	Unit
Layers								
Copper Thin Film Layer		15.45	15.86	17.81	10.78	16.04	2.62	mΩ/sq
Graphene Thin Film Layer		15.81	16.63	17.58	11.33	15.45	2.40	mΩ/sq

**Table 5:** Corrosion characterization of the current collectors.

Current Collector with a Corrosion-Resistance Layer Using Graphene Ink	
Ecorr (mV)	139.5
Icorr (uA/cm^2^)	1.19
Corrosion Rate (mmpy)	11.7 e^−6^
**Current COLLECTOR with a Corrosion-Resistance Layer Using Graphene Suspension**	
Ecorr (mV)	−68.4
Icorr (uA/cm^2^)	1.46
Corrosion Rate (mmpy)	14.4 e^−6^
**Current Collector with a Corrosion-Resistance Layer Using Graphene Dispersion**	
Ecorr (mV)	106.5
Icorr (uA/cm^2^)	0.85
Corrosion Rate (mmpy)	8.3 e^−6^
